# The baby-friendly hospital initiative and breastfeeding at birth in Brazil: a cross sectional study

**DOI:** 10.1186/s12978-016-0234-9

**Published:** 2016-10-17

**Authors:** Márcia Lazaro de Carvalho, Cristiano Siqueira Boccolini, Maria Inês Couto de Oliveira, Maria do Carmo Leal

**Affiliations:** 1 Department of Epidemiology and Quantitative Methods in Health, National School of Public Health, Oswaldo Cruz Foundation, Rua Leopoldo Bulhões, 1480, sala 806 – Manguinhos, Rio de Janeiro, CEP 21041-210 Brazil; 2 Institute of Scientific and Technological Communication and Information in Health, Oswaldo Cruz Foundation, Av. Brasil, 4.365 - Pavilhão Haity Moussatché - Manguinhos, Rio de Janeiro, CEP: 21040-900 Brazil; 3 Departament of Epidemiology and Biostatistics. Institute of Public Health, Fluminense Federal University, Rua Marques de Paraná, n° 303, anexo, 3° andar, Centro, Niterói, Rio de Janeiro, CEP: 24033-900 Brazil

**Keywords:** Breastfeeding, Maternity Hospitals, Maternal-Child Health Services, Cross-Sectional Studies, Postpartum Period, Baby-Friendly Hospital Initiative, Aleitamento materno, Maternidade, Serviços de saúde materno-infantil, Estudos seccionais, Período pós-parto, Iniciativa Hospital Amigo da Criança

## Abstract

**Background:**

Breastfeeding in the first hour after birth is important for the success of breastfeeding and in reducing neonatal mortality. Government policies are being developed in this direction, highlighting the accreditation of hospitals in the Baby-Friendly Hospital (BFH) initiative. The aim of this study was to analyze the association between delivery in a BFH (main exposure), compared to non BFH, and timely initiation of breastfeeding (outcome).

**Methods:**

Data came from the “Birth in Brazil” survey, a nationwide hospital-based study of postpartum women and their newborns, coordinated by the Oswaldo Cruz Foundation. A sample of 22,035 mothers/babies was analyzed through a hierarchical theoretical model on three levels, and all analyzes considered the complex sample design. Odds ratios were obtained by logistic regression, with a 99 % CI.

**Results:**

Among all births, 40 % occurred in hospitals accredited or in accreditation process for the BFHI and 52 % of women underwent caesarean section. In the final model, at the distal level, mothers less than 35 years old, and those who lived in the North Region, had a higher chance of timely initiation of breastfeeding. At the intermediate level, prenatal care in the public sector and advice on breastfeeding during pregnancy were directly associated with the outcome. At the proximal level, being born in a Baby-Friendly Hospital and vaginal delivery increased the chance of timely initiation of breastfeeding, while prematurity and low birth weight reduced the chance of the outcome.

**Conclusions:**

The chance of being breastfed in the first hour after birth in Baby-Friendly hospitals was twice as high as at non-accredited hospitals, which shows the importance of this initiative for timely initiation of breastfeeding.

**Electronic supplementary material:**

The online version of this article (doi:10.1186/s12978-016-0234-9) contains supplementary material, which is available to authorized users.

## Resumo

**Introdução:**

A amamentação na primeira hora de vida é importante para o sucesso do aleitamento materno e para a redução da mortalidade neonatal. Políticas governamentais vêm atuando neste sentido, destacando-se o credenciamento dos hospitais na Iniciativa Hospital Amigo da Criança (IHAC). O objetivo deste estudo é conhecer a associação entre o nascimento em Hospitais Amigos da Criança (HAC - exposição principal) e o início oportuno da amamentação (desfecho), comparado com maternidades não HAC.

**Métodos:**

Os dados vem do inquérito “Nascer no Brasil”, uma amostra de base hospitalar e abrangência nacional, sob a coordenação da Fundação Oswaldo Cruz. Foram estudados uma amostra de 22.035 mães/bebês através de um modelo teórico hierarquizado em três níveis e todas as análises consideraram o desenho complexo da amostra. As razões de chance foram obtidas por regressão logística, com IC 99 %.

**Resultados:**

Entre o total de nascimentos, 40 % ocorreram em hospitais credenciados ou em processo de credenciamento pela IHAC e 52 % das mulheres foram submetidas à cesariana. No modelo final, no nível distal, as mães com menos de 35 anos, e as que residiam na Região Norte, apresentaram uma chance maior de início oportuno da amamentação. No nível intermediário, a realização de pré-natal no setor público e a orientação sobre amamentação tiveram associação direta com o desfecho. No nível proximal, ter nascido em Hospital Amigo da Criança e via de parto normal aumentaram a chance do início oportuno da amamentação, enquanto ser bebê prematuro e apresentar baixo peso ao nascer reduziram a chance do desfecho.

**Conclusões:**

A chance de uma criança ser amamentada na primeira hora de vida nos hospitais amigos da criança foi duas vezes maior do que nos hospitais não credenciados, o que mostra a importância dessa iniciativa para o início oportuno da amamentação.

## Background

The World Health Organization recommends breastfeeding in the first hour after birth as part of the Baby-Friendly Hospital Initiative (BFHI) strategy in order to reduce neonatal mortality [1, 2] and to improve breastfeeding duration [3, 4]. The contact with human milk produced in the first days of life (colostrum), promotes intestinal colonization with saprophytes [5] and enhances the newborn immune system by providing oligosaccharides, immunoglobulin-A and other immune compounds [6]. Brazilian’s Ministry of Health has adopted BFHI as part of its breastfeeding promotion, protection and support policy, having 335 hospitals accredited in this policy in 2010 (http://www.unicef.org/brazil/pt/br_listaIHAC2010.pdf).

Despite the importance of timely initiation of breastfeeding, several barriers to this practice have been identified [7, 8] including mother and hospital related barriers. Among them, caesarean section and hospital practices have major importance, since mothers have little or no power to decide if they are going to breastfeed or not their newborns [8].

Since the Baby-Friendly Hospital Initiative may play a key role in promoting timely initiation of breastfeeding, this study aimed to identify the association between delivery at a Baby-Friendly Hospital and breastfeeding at the first hour of life.

## Methods

This was a hospital-based cross sectional study, with a complex sample to represent all births that occurred in hospitals with more than 500 deliveries/year in Brazil (which correspond to 78.6 % of all hospital births), with field work conducted from February 2011 to October 2012. This study, named “Birth in Brazil: national survey into labor and birth”, was coordinated by the Oswaldo Cruz Foundation and the sample was based on the National Information System [9].

The sample design was selected in three stages: in the first stage, hospitals were stratified according to the five Brazilian Regions (North, Northeast, Southeast, Midwest and South), location (state capital and other cities), and type of hospital funding (public, mixed or private), with a total of 30 strata. In this stage 266 hospitals were selected with probability of selection proportional to the number of deliveries in each strata. In the second stage, the number of days needed to interview 90 puerperal women in each hospital was selected by inverse sampling method. In the third stage, the women eligible on each day of the fieldwork were selected. Sample losses due to refusal to participate or hospital discharge were replaced by selecting other puerperal women at the same hospital.

The inclusion criteria to the “Birth in Brazil” survey was: hospital live births with gestational age of more than 22 weeks recorded in the medical file or weight greater than 500 g. All miscarriages were excluded. The sample size was based on a caesarean delivery rate of 46.6 %, to detect differences of 14 % between hospitals, with an alpha of 5 % and power of 95 %, having a minimum of 341 puerperal mothers in each strata.

In total, interviews were conducted with 23,940 women, among 266 hospitals distributed in 191 municipalities, covering all the 27 Brazilian states. Trained field researchers interviewed mothers with an electronic questionnaire in the first 24 h post partum. The questions were related to individual and gestational characteristics, prenatal and delivery care, neonatal characteristics, and breastfeeding at birth. A different questionnaire was applied to the hospital manager. More details about the sample design and field work can be obtained [10].

This study was approved by the Research Ethics Committee of ENSP/FIOCRUZ, under the report n°. 92/2010. Every care was taken towards ensuring privacy and confidentiality of the information. Before each interview was conducted, the interviewee’s consent was obtained, after reading the free and informed consent statement.

The present study investigated the factors associated with breastfeeding at first hour of life (outcome), also denominated ‘timely breastfeeding initiation’, which has been categorized in a dichotomous way (yes, no) based on questions regarding breastfeeding at delivery room and time to initiate breastfeeding. Based on potential conditions that may impede or obstruct breastfeeding at first hour, we have established the following exclusion criteria: mothers tested positive for HIV (according to medical records) and/or with *near missing* condition [11]; babies who died in the neonatal period; with APGAR < 7 at 5^th^ minute of life; with birth weight <1500 g; and/or gestational age < 32 weeks. In addition, 924 (around 4 %) mothers did not know/answer the questions about breastfeeding initiation, resulting in a final sample of 22,035 mothers and their respective babies.

The exposure variable of being born in a Baby Friendly Initiative Hospital (divided in three categories: yes, in process, and no) was obtained from an interview with the hospital manager and attributed to each mother that had a delivery in that hospital.

**Fig. 1:**
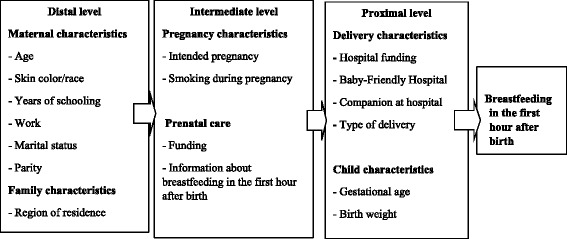
Hierarchical theoretical model to analyze breastfeeding in the first hour after birth

Based on a recent literature review [7] and in a conceptual framework [12], we arranged the confounding variables in a hierarchic model, in three distinct levels based on their proximity to the outcome: distal – mother and family socioeconomic characteristics; intermediate – pregnancy and prenatal characteristics; and proximal – related to delivery conditions and newborn characteristics (Fig. 1).

It is important to state that in Brazil we classify race/ethnicity not based on ancestry taxonomy, but based on self-reported skin color/race, according to the official Brazilian Statistic Institute ‘Instituto Brasileiro de Geografia e Estatística’ definitions, 2010.

All analysis has considered the complex sample design, having the mothers that breastfed their children on the first hour of life as reference, implying to interpret the results as the chance to breastfed in the first hour after birth. Initially we estimated the Chi-square test for each variable and the outcome and obtained the unadjusted Odds Ratio (OR) and the 99 % Confidence Interval (99 % CI). To avoid residual confounding, we selected all variables with *p*-value ≤ 0.20 to compose the modeling process.

In sequence, we estimated logistic regression models, with 99 % CI, following the hierarchic model (Fig. 1) in three steps: first, adjusting all the distal variables together and removing the ones without statistical significance; second, adjusting the intermediate variables along with the remaining distal variables and removing the intermediate ones that did not achieved *p* < 0.01; third, adjusting all the proximal variables with the remaining ones from the previous steps – only the variables with *p*-value <0.01 were retained.

## Results

We found that among children born in hospitals with more than 500 deliveries/year in Brazil, 56 % were breastfed in the first hour after birth, when considering only mothers who were able to breastfeed and newborns with suckling conditions. In this study, around one mother in four did not finish the elementary schooling, more than half were primiparous, and almost all received prenatal care. When examining the delivery characteristics, 40 % had their babies in Baby- Friendly Hospitals or in accreditation process, 52 % were submitted to caesarean section delivery, and 8.7 % had premature babies with gestational week varying from 32 0/7 and 36 6/7 (Table 1).

**Table 1:** Prevalence of maternal, hospital and child characteristics, Brazil, 2011

Variable/categories	Number^a^	Percent^b^	99 % CI^b^
Maternal age			
12–19 years	4170	18.8	17.7–20.1
20–34 years	15454	70.9	69.7–72.1
35 years or more	2232	10.2	9.4–11.1
Missing	5		
Skin color/Race^c^			
White	7486	34.3	31.9–36.9
Black	1835	8.4	7.3–9.7
Brown	12150	55.7	53.3–58.2
Yellow	233	1.1	0.8–1.5
Indigenous	91	0.4	0.2–0.7
Missing	4		
Maternal years of schooling^d^			
< = 7	5572	25.7	23.8–27.7
8 to 10	5566	25.7	24.4–27.0
11 to 14	88556	39.4	37.1–41.8
15 or more	2001	9.2	7.8–10.9
Missing	103		
Maternal work			
No	12885	59.1	57.4–60.8
Yes	8913	40.9	39.2–42.6
Missing	1		
Marital status at delivery			
Without companion/spouse	3984	18.3	17.2–19.4
With companion/spouse	17808	81.7	80.6–82.8
Missing	6		
Parity			
Primiparous	11618	53.3	52.0–54.6
Multiparous	10180	46.7	45.4–48.0
Missing	1		
Brazilian region of residence			
North	2106	9.7	8.6–10.9
Northeast	6160	28.3	26.0–30.6
Southeast	9318	42.7	39.7–45.8
South	2775	12.7	11.5–14.0
Middle West	1440	6.6	5.5–7.9
Missing	0		
Intended pregnancy			
Yes	9619	44.4	43.0–45.8
Did not intend to get pregnant now	5619	25.9	24.7–27.2
Not at all	6420	29.6	28.2–31.1
Missing	140		
Funding of prenatal care			
No prenatal care	233	1.1	0,8–1.4
Public	15065	69.3	67.1–71.4
Mixed	825	3.8	3.3–4.4
Private	5626	25.9	24.0–27-9
Missing	49		
Information about breastfeeding at prenatal care			
No prenatal care	233	1.1	0.8–1.4
Yes	13848	35.0	33.1–36.9
No	7624	63.5	61.6–65.4
Missing	93		
Smoking during pregnancy			
No	19712	90.5	89.6–91.3
Yes, part of pregnancy	607	2.8	2.4–3.3
Yes, whole pregnancy	1467	6.7	6.1–7.4
Missing	13		
Hospital funding			
Public	17272	79.2	77.2–81.1
Private	4527	20.8	18.9–22.8
Missing	0		
Baby-Friendly Hospital^e^			
Yes	7159	32.8	25.7–40.9
In process	1571	7.2	3.7–13.5
No	13068	60.0	51.9–67.5
Missing	0		
Companion at hospital			
None	5201	23.9	20.0–28.2
Part time companion	12171	55.9	51.3–60.3
Full time companion	4418	20.3	16.5–24.6
Missing	8		
Type of delivery			
Normal	10249	47.0	44.0–50.0
Forceps	312	1.4	0.9–2.3
Intrapartum caesarean	1766	8.1	6.9–9.4
Antepartum caesarean	9472	43.5	40.9–46.1
Missing	0		
Gestational age			
32 0/7 to 36 6/7 weeks	1905	8.7	7.7–10.0
37 0/7 or more	19894	91.3	90.0–92.3
Missing	0		
Birth weight			
2500 g or more	20181	93.4	92.5–94.2
1500–2499 g	1422	6.6	5.8–7.5
Missing	195		

^a^Final valid sample of selected mother-child that answered the ‘Birth in Brazil’ questionnaire in 2011, unweighted cases

^b^Final valid sample prevalence and 99 % Confidence Interval (99 % CI), considering the complex sample design

^c^Self-reported skin color/race, according to Brazilian classification based on ‘Instituto Brasileiro de Geografia e Estatística’definitions, 2010

^d^Based on ‘Instituto Brasileiro de Geografia e Estatística’definitions, 2010

^e^Based on hospital manager information

In the bivariate analysis, we found an association (*p* < 0.20) between timely initiation of breastfeeding and the following distal variables: maternal age, skin color/race, maternal years of schooling, maternal work, marital status at delivery, parity, and Brazilian region of residence. Considering the intermediate variables, we found an association with prenatal care funding and information about breastfeeding at prenatal care. The proximal variables associated with the outcome were hospital funding, Baby-Friendly Hospital, type of delivery, gestational age and birth weight (Table 2).

**Table 2:** Unadjusted factors associated with breastfeeding in the first hour after birth, according to mother, newborn and hospital characteristics, Brazil, 2011

Variable/categories	Prevalence^a^	Prevalence 99 % CI^a^	Unadjusted OR^b^	Unadjusted OR 99 % CI^b^
Maternal age				
12–19 years	62.5	58.2–66.6	2.01	1.60–2.52
20–34 years	55.8	51.8–59.8	1.52	1.29–1.81
35 years or more	45.3	40.1–50.7	1.00	-
Skin color/race^c^				
White	51.4	46.6–56.2	1.00	-
Black	59.4	53.6–64.9	1.38	1.07–1.79
Brown	58.1	54.0–62.1	1.31	1.11–1.55
Yellow	62.6	48.7–74.7	1.58	0.90–2.78
Indigenous	75.7	53.4–89.8	2.94	1.03–8.41
Maternal years of schooling^d^				
< = 7	62.8	58.7–66.7	2.72	2.08–3.55
8 to 10	61.4	57.1–65.7	2.57	1.96–3.36
11 to 14	52.2	47.6–56.9	1.76	1.41–2.19
15 or more	38.3	32.6–44.3	1.00	-
Maternal work				
No	60.0	56.2–63.8	1.49	1.31–1.70
Yes	50.2	45.8–54.6	1.00	-
Marital status at delivery				
Without companion/spouse	59.0	54.3–63.4	1.16	1.02–1.31
With companion/spouse	55.4	51.5–59.1	1.00	-
Parity				
Primiparous	58.6	54.7–62.4	1.00	-
Multiparous	53.1	48.9–57.3	1.25	1.11–1.50
Brazilian region of residence				
North	71.0	65.3–76.1	2.30	1.56–3.39
Northeast	53.7	47.6–59.7	1.09	0.75–1.59
Southeast	51.5	44.4–58.6	1.00	-
South	61.1	50.7–70.6	1.48	0.89–2.47
Middle West	63.1	55.7–70.0	1.61	1.06–2.46
Intended pregnancy				
Yes	54.0	49.9–58.1	1.00	-
Did not intend to get pregnant now	56.7	52.2–61.1	1.17	0.98–1.26
Not at all	58.6	54.2–62.7	1.20	1.04–1.40
Smoking during pregnancy				
No	55.6	51.8–59.3	1.00	-
Yes, part of pregnancy	57.3	49.0–65.3	1.07	0.81–1.43
Yes, whole pregnancy	61.0	54.7–66.9	1.25	1.01–1.55
Funding of prenatal care				
No prenatal care	52.8	40.2–65.0	1.71	0.98–2.99
Public	62.4	58.3–66.3	2.55	1.97–3.29
Mixed	52.7	45.3–60.0	1.71	1.23–2.38
Private	39.5	33.7–45.6	1.00	-
Information about breastfeeding at prenatal care				
No prenatal care	52.8	40.2–65.0	1.02	0.61–1.71-
Received	58.2	54.5–61.8	1.28	1.11–1.48
Did not receive	52.2	47.2–57.0	1.00	-
Hospital funding^e^				
Public	61.1	57.0–65.1	2.73	1.94–3.86
Private	36.5	29.4–44.3	1.00	-
Baby-Friendly Hospital^e^				
Yes	69.4	64.9–73.6	2.49	1.85–3.34
In process	63.9	51.0–75.1	1.94	1.11–3.41
No	47.7	42.5–53.0	1.00	-
Companion at hospital				
None	55.6	49.8–61.2	1.00	-
Part time companion	54.3	49.8–58.8	0.95	0.75–1.20
Full time companion	61.1	55.4–66.6	1.26	0.92–1.72
Type of delivery				
Normal	70.3	66.2–74.1	3.29	2.67–4.06
Forceps	60.3	47.9–71.5	2.11	1.27–3.51
Intrapartum caesarean	48.7	43.0–54.4	1.32	1.07–1.63
Antepartum caesarean	41.8	37.2–46.5	1.00	-
Gestational age				
32 0/7 to 36 6/7 weeks	37.4	32.9–42.1	0.44	0.36–0.53
37 0/7 or more	57.8	53.9–61.6	1.00	-
Birth weight				
1500–2499 g	57.4	53.5–61.2	0.42	0.36–0.50
2500 g or more	36.3	31.0–42.0	1.00	-
Total	56.0	52.2–59.7		

^a^Final valid sample prevalence and 99 % Confidence Interval (99 % CI), considering the complex sample design

^b^Unadjusted Odds Ratio (OR) and 99 % CI, considering the complex sample design

^c^Self-reported skin color/race, according to Brazilian classification based on ‘Instituto Brasileiro de Geografia e Estatística’definitions, 2010

^d^Based on ‘Instituto Brasileiro de Geografia e Estatística’definitions, 2010

^e^Based on hospital manager information

In the final adjusted model, mothers with higher likelihood to breastfeed in the first hour after birth were less than 35 years old, residents at the North Region of Brazil (compared to the Southeast region), with prenatal care at the public sector, and who received information about breastfeeding at the first hour of life in this period. The mothers that gave birth at a Baby-Friendly Hospital and with vaginal delivery also had a higher odds to timely initiation of breastfeeding. Low birth weight and premature babies had lower odds of being breastfed in the first hour after birth (Table 3).

**Table 3:** Factors associated with breastfeeding in the first hour after birth in the final adjusted model, Brazil, 2011

Variable/categories	AOR^a^	99 % Confidence Interval^a^
Maternal age		
12–19 years	1.33	(1.02–1.74)
20–34 years	1.23	(1.01–1.50)
35 years or +	1.00	-
Brazilian region of residence		
North	1.81	(1.19–2.76)
Northeast	0.92	(0.63–1.35)
Middle West	1.43	(0.83–2.46)
South	1.37	(0.81–2.32)
Southeast	1.00	-
Funding of prenatal care		
No prenatal care	0.96	(0.20–4.74)
Public	1.33	(1.01–1.76)
Mixed	1.35	(0.92–1.99)
Private	1.00	-
Information about breastfeeding at prenatal care		
No prenatal care	1.03	(0.30–3.56)
Yes	1.38	(1.17–1.63)
No	1.00	-
Baby-Friendly Hospital^b^		
Yes	2.07	(1.50–2.86)
In process	1.44	(0.71–2.93)
No	1.00	-
Type of delivery		
Normal	2.81	(2.19–3.62)
Forceps	2.11	(1.30–3.44)
Intrapartum caesarean	1.18	(0.94–1.48)
Antepartum caesarean	1.00	-
Gestational age		
32 0/7 to 36 6/7 weeks	0.47	(0.38–0.59)
37 0/7 or more	1.00	-
Birth weight		
1500–2499 g	0.51	(0.39–0.66)
2500 g or more	1.00	-

^a^Adjusted Odds Ratio (AOR) and 99 % CI, obtained from a logistic regression model and considering the complex sample design, adjusted for educational level and parity

^b^Based on hospital manager information

## Discussion

More than half (56 %) of the babies born in Brazil in 2011–12, with conditions that allowed breastfeeding, were timely breastfed, which represents an improvement compared to the 43 % timely breastfed babies observed in the National Survey of Demography and Health conducted in 2006 (PNDS, 2006). However, the results obtained were below those found in a survey held in 2008 in the Brazilian capitals, where 67 % of the babies were breastfed in the first hour after birth [13]. This disparity may be due to methodological differences and to the sample strategy, as the 2008 survey was conducted only in capitals and with children under one year old, while the current “Birth in Brazil” survey had a broader sample and interviewed mothers in the first day after birth, decreasing the possibility of recall bias.

Several breastfeeding indicators have improved in Brazil since the National Breastfeeding Program was released by the Ministry of Health in 1981 [14]. However, only the indicator “breastfeeding in the first hour after birth” achieved the WHO status of “good” (between 50–89 % - MS, 2009). The WHO recommends putting the babies in skin-to-skin contact soon after delivery, giving mothers support to start breastfeeding within this sensitive period [15], since the newborn has the reflex to search by himself the mother’s areola [16].

Among all Brazilian deliveries, four in ten occurred in Baby-Friendly Hospitals (33 %) or in those in process to become BFH (7 %), which represents a great improvement compared to 2004, when only one in four deliveries occurred in BFH [17]. In Brazil, in 2011, the chance of being breastfed in the first hour after birth doubled if the child was born in a BFH.

A similar effect was observed in a maternity hospital in the accreditation process to become BFH in the South of Brazil [18], corroborating the importance of BFH accreditation to improve not only timely breastfeeding, but also exclusive breastfeeding duration among healthy newborns [19] and those that need intensive care treatment [20], as well as to reduce pacifier use [21].

Although WHO recommends a caesarean section delivery rate of 10 % [22], our study found a c-section rate of 52 %. These rates differed between BFH (40.6 %) and non BFH (58.9 %), which may be explained by an additional criteria established by the Brazilian Ministry of Health for certification as a Baby-Friendly Hospital (besides the UNICEF/WHO requirements of compliance with the 10 steps): the reduction of caesarean section rates [23]. In the “Birth in Brazil” study, children born by vaginal delivery had almost a three times chance of being breastfed in the first hour after birth than those born by c-section. This is consistent with the results of a study conducted in Rio de Janeiro, where c-section reduced by half the prevalence of breastfeeding in the first hour after birth [8], and with the findings of a systematic review: c-section is the most frequent factor negatively associated with timely breastfeeding [7], since post partum procedures and routines may delay the early contact between mother and baby.

Moreira et al. [24] reported a synergic effect of vaginal delivery and Baby-Friendly Hospitals, as both represent good hospital practices to the newborn. We assume that caesarean section should not represent a risk to timely breastfeeding if both newborns and mothers are in good condition if the surgery occurs after initiation of labor signs, which could indicate the newborn maturity to initiate breastfeeding. However, the caesarean rate in Brazil is significantly higher in the private sector, with approximately 80 % of caesarean sections carried out without the mother having gone into labor [25].

Both premature and low birth weight newborns had half the chance of being breastfed in the first hour after birth. This can be due to unnecessary routines and interventions (oxygen therapy, aspirating the superior airways, among others) that may unnecessarily separate mother and child in the delivery room [24]. Our study only included premature newborns with 32 0/7 to 36 6/7 gestational weeks, and low birth weight children between 1500 and 2499 g, conditions which, besides inspiring extra care, may not be a barrier to breastfeeding in the first hour after birth. It is important to improve both prematurity prevention and neonatal care to the most vulnerable children [26], since another study also showed prematurity as a risk factor to breastfeeding in the first hour after birth [27].

Mothers that received information about breastfeeding during prenatal care had higher chances to breastfed their babies in the first hour after birth, similar to the result of a study conducted in Bahia [27] (Vieira, 2010), showing the importance of prenatal care to breastfeeding initiation.

Considering distal factors, only mother’s age and region of residence were associated with the outcome. A study in the South of Brazil also found an association between mother’s age above 34 years and lower chances of breastfeeding in the first hour after birth [18]. This result could be due to a cohort effect, as older mothers were less exposed to the increasing practice of breastfeeding at birth in Brazil [28]. As to the region of residence, a national survey conducted in 2006 [28] and a study in the Brazilian capitals in 2008 [13] evidenced a higher prevalence of breastfeeding in the first hour in the North region of Brazil, what can be due to cultural factors, since most of the indigenous population are concentrated in this region.

## Conclusions

The studied proximal factors were the most strongly associated with timely breastfeeding, bringing evidence about the importance of adopting Baby-Friendly Hospital Initiative to improve perinatal practices and timely breastfeeding initiation. Special attention should be given to the negative association found between caesarean section delivery without clinical indication and breastfeeding in the first hour after birth, bringing more evidence to the ongoing government efforts to diminish this harmful practice in Brazil. Prematurity and low birth weight are factors difficult to be modified, but gains in prenatal care access and quality could contribute to a decline on their prevalence and to improve timely breastfeeding rates. We recommend the reinforcement of BFHI implementation, extending this initiative to the private sector. This measure could contribute not only to improve timely breastfeeding rates, but also reducing unnecessary caesarean section delivery.

### Portuguese version

A Portuguese translation of this article is available as Additional file 1.

### Peer review

The reviewer reports for this article are available as Additional file 2.

## Additional files

Additional file 1: Portuguese version. (DOCX 82 kb)
Additional file 2: Peer review. (DOCX 28 kb)

## Abbreviations

APGAR: Refers to some organic parameters considered in the first minutes of life: A - Activity; P - Pulse ; G - Grimace (reflex readiness); A - Appearance (skin color); R - Respiration
BFH: Baby-Friendly Hospital
BFHI: Baby-Friendly Hospital Initiative
CI: Confidence interval
ENSP: National School of Public Health
FIOCRUZ: Oswaldo Cruz Foundation
HIV: Human immunodeficiency virus
OR: Odds ratio
UNICEF: United Nations International Children’s Emergency Fund
WHO: World Health Organization
