# Supplementary material for: The baby-friendly hospital initiative and breastfeeding at birth in Brazil: a cross sectional study
Source: Reprod Health. 2016 Oct 17;13(Suppl 3):119. doi: 10.1186/s12978-016-0234-9 (PMC5073809; doi:10.1186/s12978-016-0234-9)
Supplement: Additional file 1: — Portuguese version. (DOCX 82 kb) [file 12978_2016_234_MOESM1_ESM.docx]

**PÁGINA DE ROSTO**

**Título: "Os Hospitais Amigos da Criança e a Amamentação na Primeira Hora de vida: um estudo seccional”**

**Autores:**

**Márcia Lazaro de Carvalho (autor para correspondência)**

Departamento de Epidemiologia e Métodos Quantitativos em Saúde. Escola Nacional de Saúde Pública. Fundação Oswaldo Cruz, Rio de Janeiro, Brasil. Rua Leopoldo Bulhões, 1480, sala 806 - Manguinhos, Rio de Janeiro – CEP 21041-210. Email: [marcialazaroc@gmail.com](mailto:marcialazaroc@gmail.com). Telefone: +55 (21) 2598-2623

**Cristiano Siqueira Boccolini**

Instituto de Comunicação e Informação Científica e Tecnologia em Saúde, Fundação Oswaldo Cruz, Rio de Janeiro, Brasil. Av. Brasil, 4.365 - Pavilhão Haity Moussatché - Manguinhos, Rio de Janeiro - CEP: 21040-900

**Maria Inês Couto de Oliveira**

Departamento de Epidemiologia e Bioestatística. Instituto de Saúde Coletiva, Universidade Federal Fluminense. Rua Marques de Paraná, nº 303, anexo, 3º andar, Centro, Niterói, Rio de Janeiro, Brasil. CEP: 24033-900. Email: [marinesco@superig.com.br](mailto:marinesco@superig.com.br). Telephone: +55(21) 2629-9342

**Maria do Carmo Leal**

Departamento de Epidemiologia e Métodos Quantitativos em Saúde. Escola Nacional de Saúde Pública. Fundação Oswaldo Cruz, Rio de Janeiro, Brasil. Rua Leopoldo Bulhões, 1480, sala 809 - Manguinhos, Rio de Janeiro – CEP 21041-210.

**Título: "Os Hospitais Amigos da Criança e a Amamentação na primeira Hora de vida: um estudo seccional.”**

**Resumo**

**Introdução**: A amamentação na primeira hora de vida é importante para o sucesso do aleitamento materno e para a redução da mortalidade neonatal. Políticas governamentais vêm atuando neste sentido, destacando-se o credenciamento dos hospitais na Iniciativa Hospital Amigo da Criança (IHAC). O objetivo deste estudo é conhecer a associação entre o nascimento em Hospitais Amigos da Criança (HAC – exposição principal) e a amamentação na primeira hora de vida (desfecho), comparado com maternidades não HAC. **Métodos**: Os dados vem do inquérito “Nascer no Brasil”, uma amostra de base hospitalar e abrangência nacional, sob a coordenação da Fundação Oswaldo Cruz. Foi estudada uma amostra de mães/bebês (n= 22.035) por meio de um modelo teórico hierarquizado em três níveis, considerando o desenho complexo da amostra. As razões de chance foram obtidas por regressão logística, com intervalo de confiança de 99%. **Resultados**: Do total de nascimentos, 40% ocorreram em hospitais credenciados ou em processo de credenciamento pela IHAC. No modelo final, no nível distal, as mães com menos de 35 anos, e as que residiam na Região Norte, apresentaram uma chance maior de início oportuno da amamentação. No nível intermediário, a realização de pré-natal no setor público e a orientação sobre amamentação tiveram associação direta com o desfecho. No nível proximal, ter nascido em Hospital Amigo da Criança e via de parto normal aumentaram a chance do início oportuno da amamentação, enquanto ser bebê prematuro e apresentar baixo peso ao nascer reduziram a chance do desfecho. **Conclusões**: A chance de uma criança ser amamentada na primeira hora de vida nos hospitais amigos da criança foi duas vezes maior que nos hospitais não credenciados, o que mostra a importância dessa iniciativa para o início oportuno da amamentação.

**Palavras-chave: Aleitamento materno, maternidade, serviços de saúde materno-infantil, estudos seccionais, período pós-parto, Iniciativa Hospital Amigo da Criança.**

Introdução

A Organização Mundial de Saúde recomenda a amamentação na primeira hora após o nascimento, como parte da estratégia Iniciativa Hospital Amigo da Criança (IHAC) para reduzir a mortalidade neonatal [1, 2] e melhorar a duração da amamentação[3, 4]. O contato com o leite humano produzido nos primeiros dias de vida (colostro), promove a colonização intestinal com bactérias saprófitas [5] e melhora o sistema imunológico do recém-nascido fornecendo oligossacarídeos, imunoglobulina-A e outros componentes imunológicos [6]. O Ministério da Saúde do Brasil adotou a estratégia Iniciativa Hospital Amigo da Criança (IHAC) como parte de sua política de promoção proteção e apoio ao aleitamento materno, tendo 335 hospitais credenciados nesta política em 2010 ([http://www.unicef.org/brazil/pt/br_listaIHAC2010.pdf).](http://www.microsofttranslator.com/bv.aspx?from=&to=pt&a=http://www.unicef.org/brazil/pt/br_listaIHAC2010.pdf).)

Apesar da importância do início oportuno da amamentação, foram identificados vários obstáculos a esta prática [7, 8], incluindo a cesariana e as práticas hospitalares, uma vez que as mães têm pouco ou nenhum poder para decidir se elas vão ou não amamentar seus recém-nascidos [8].

Como a Iniciativa Hospital Amigo da Criança pode desempenhar um papel fundamental na promoção do início oportuno do aleitamento materno, este estudo objetivou identificar a associação entre o nascimento em um Hospital Amigo da Criança e amamentação na primeira hora de vida.

Métodos

Este foi um estudo seccional de base hospitalar com uma amostragem complexa para representar todos os nascimentos ocorridos em hospitais com mais de 500 nascimentos/ano no Brasil (que correspondem a 78,6% de todos os nascimentos hospitalares), com trabalho de campo realizado de fevereiro de 2011 a outubro de 2012. Este estudo, denominado "Nascer no Brasil: pesquisa nacional sobre parto e nascimento", foi coordenado pela Fundação Oswaldo Cruz e a amostra baseou-se em dados obtidos do Sistema de Informação de Nascidos Vivos [9].

O desenho da amostra se deu em três estágios: no primeiro estágio, os hospitais foram estratificados de acordo com as cinco regiões brasileiras (Norte, Nordeste, Sudeste, Centro-Oeste e Sul), localização (capital do estado e outras cidades) e tipo de financiamento do hospital (público, misto ou privado), com um total de 30 estratos. Nesta etapa, 266 hospitais foram selecionados com probabilidade de seleção proporcional ao número de partos em cada estrato. No segundo estágio, o número de dias necessários para entrevistar 90 mulheres no período puerperal em cada hospital, foi estabelecido pelo método de amostragem inversa. No terceiro estágio, foram selecionadas as mulheres elegíveis em cada dia de trabalho de campo. As perdas devido à recusa em participar ou alta hospitalar foram substituídas, selecionando outras mulheres no período puerperal no mesmo hospital.

Os critérios de inclusão para a pesquisa “Nascer no Brasil” foram: nascimentos hospitalares com idade gestacional de mais de 22 semanas registrada no prontuário médico ou peso superior a 500 gramas. Foram excluídos todos os abortos. O tamanho da amostra foi baseado em uma taxa de cesariana de 46,6%, para detectar diferenças de 14% entre os hospitais, com um valor de p de 5% e poder de teste de 95%, tendo um mínimo de 341 mães em cada estrato. No total, foram realizadas entrevistadas 23.940 mulheres, em 266 hospitais distribuídos em 191 municípios, cobrindo todos os 27 estados brasileiros. Pesquisadores de campo treinados entrevistaram as mães utilizando um questionário eletrônico nas primeiras 24 horas após o parto.

As perguntas eram relativas a características individuais e gestacionais, à assistência

pré-natal, ao parto e a características do neonato, e ao aleitamento materno ao nascimento. Para o diretor do hospital, foi aplicado um questionário diferente. Mais detalhes sobre o trabalho de campo e projeto de amostra podem ser obtidas em publicações prévias[10].

Este estudo foi aprovado pelo Comitê de Ética e Pesquisa da ENSP/FIOCRUZ, sob o nº de relatório 92/2010. Todo cuidado foi tomado para garantir a privacidade e confidencialidade da informação. Antes de cada entrevista, foi obtido o consentimento da entrevistada, depois de ler as instruções do instrumento de livre e consentimento.

O presente estudo investigou os fatores associados à amamentação na primeira hora de vida (desfecho), também denominado 'início oportuno do aleitamento materno', categorizado de forma dicotômica (sim, não) baseado em perguntas sobre aleitamento materno na sala de parto e o tempo para iniciar a amamentação. Com base em condições potenciais que podem impedir ou dificultar a amamentação na primeira hora, foram estabelecidos os seguintes critérios de exclusão: mães com sorologia positiva para o HIV (de acordo com registros médicos) e/ou com condição *de near missing* [11]; bebês que morreram no período neonatal; com APGAR < 7 no 5º minuto de vida; com peso de nascimento < 1500 gramas; e/ou < 32 semanas de idade gestacional. Além disso, 924 (cerca de 4%) mães não sabiam/não responderam às perguntas sobre o início da amamentação, resultando em uma amostra final de 22.035 mães e seus respectivos bebês.

A variável de exposição “nascer em um Hospital Amigo da Criança (HAC)” (dividida em três categorias: sim; em processo de credenciamento e não) foi obtida a partir de uma entrevista com o diretor do hospital.

Baseado em uma recente revisão de literatura [7] e em um quadro teórico conceitual [12], apresentamos as variáveis de confundimento em um modelo hierarquizado, em três níveis distintos, com base na sua proximidade com o desfecho: distal- características socioeconômicas da mãe e família; intermediário – características da gravidez e pré-natal; e proximal – relacionados às condições do parto e características do recém-nascido (Figura 1).

INSERIR FIGURA 1 AQUI

É importante declarar que, no Brasil, classificamos raça/etnicidade, não com base na taxonomia da ancestralidade, mas com base na cor da pele/raça auto-relatados, de acordo com as definições oficiais do Instituto Brasileiro de Geografia e Estatística (IBGE), 2010. Toda a análise considerou o desenho de amostra complexa, tendo as mães que amamentaram seus filhos na primeira hora de vida, como referência, implicando na interpretação dos resultados como a chance de amamentar na primeira hora após o nascimento. Inicialmente estimou-se o teste qui-quadrado para cada variável e o desfecho e obteve-se a Odds Ratio (OR) não ajustada e intervalo de confiança de 99% (IC 99%). Para evitar confusão residual, selecionamos todas as variáveis com valor de p ≤ 0,20 para compor o processo de modelagem.

Em sequência, estimamos um modelo de regressão logística, com IC 99%, segundo modelo hierarquizado (Figura 1) em três estágios: primeiro, todas as variáveis distais foram ajustadas ao mesmo tempo e removidas as sem significância estatística; segundo, as variáveis intermediárias foram ajustadas juntamente com as variáveis distais que permaneceram no modelo e foram removidas as variáveis intermediárias que não alcançaram valor de p < 0,01; terceiro, foram ajustadas todas as variáveis proximais com as variáveis restantes das etapas anteriores – e somente as variáveis com valor de p < 0,01 foram retidas.

Resultados

Entre as crianças nascidas em hospitais com mais de 500 partos/ano no Brasil, 56% foram amamentadas na primeira hora após o nascimento, sendo consideradas nessa análise apenas as mães capazes de amamentar e os recém-nascidos com condições de sugar o leite materno. Neste estudo, cerca de uma mãe em cinco não terminou o ensino elementar, mais da metade eram primíparas, e quase todas receberam cuidados pré-natais. Ao examinar as características do parto, 40% tiveram seus bebês em Hospitais Amigos da Criança, 45% foram submetidas à cesariana e 8,7% tiveram bebês prematuros, com semana gestacional variando de 32 0/7 a 36 6/7 (Tabela 1).

Na análise bivariada, foi encontrada uma associação (p < 0,20) entre o início oportuno da amamentação e as seguintes variáveis distais: idade materna, cor da pele/raça , anos de escolaridade materna, trabalho materno, estado civil na ocasião do parto, paridade e região brasileira da residência. Considerando as variáveis intermediárias, foi encontrada associação com financiamento do pre-natal e informação sobre amamentação no pré-natal. As variáveis proximais associadas ao desfecho foram financiamento do hospital, nascimento em Hospital Amigo da Criança, tipo de parto, idade gestacional e peso ao nascer (Tabela 2).

No modelo final ajustado, as mães com maior chance de amamentar na primeira hora após o nascimento tinham menos de 35 anos de idade, eram residentes da Região Norte do Brasil (comparado à Região Sudeste), tinham feito pré-natal no setor público, e tinham recebido informações no pré-natal sobre amamentação na primeira hora de vida. As mães que deram à luz em um hospital Amigo da Criança e por via de parto vaginal também tiveram uma maior chance de iniciar o aleitamento materno de maneira oportuna. Bebês com baixo peso ao nascer e prematuros tiveram menor chance de serem amamentados na primeira hora após o nascimento (Tabela 3).

Inserir TABELA 1, 2 E 3 aqui

Discussão

Mais da metade (56%) dos bebês nascidos no Brasil em 2011-12, em condições que permitiam a amamentação, foram amamentados de maneira oportuna , o que representa uma melhoria em comparação com os 43% de bebês amamentados ao nascimento, observados na pesquisa nacional de demografia e saúde realizada em 2006 (PNDS, 2006). No entanto, os resultados obtidos foram inferiores aos encontrados em uma pesquisa realizada em 2008 nas capitais brasileiras, onde 67% dos bebês foram amamentados na primeira hora após o nascimento [13]. Esta disparidade pode ser devida a diferenças metodológicas e à estratégia de amostragem. A pesquisa de 2008 foi realizada apenas nas capitais e com as crianças de menos de um ano de idade, enquanto a atual pesquisa "Nascer no Brasil" tinha uma amostra mais ampla e entrevistou mães no primeiro dia após o nascimento, diminuindo a possibilidade de viés de memória.

Vários indicadores de aleitamento materno têm melhorado no Brasil, desde que o Programa Nacional de Aleitamento Materno foi lançado pelo Ministério da Saúde em 1981 [14]. No entanto, apenas o indicador "amamentação na primeira hora após o nascimento" alcançou o status de "bom" pela OMS (entre 50-89% - MS, 2009). A OMS recomenda colocar os bebês em contato pele a pele logo após o parto, dando suporte às mães para iniciar a amamentação durante esse período sensível [15], já que o recém-nascido tem o reflexo de busca pela aréola da mãe [16].

Entre todos os partos brasileiros, quatro em cada dez ocorreram em hospitais Amigos da Criança (33%) ou em hospitais em processo de credenciamento na IHAC (7%), o que representa uma grande melhoria comparado ao ano de 2004, quando apenas um em cada quatro partos ocorreu em HAC [17]. No Brasil, em 2011, a chance de ser amamentado na primeira hora após o nascimento dobrou se a criança nasceu em um HAC.

Um efeito similar foi observado em uma maternidade em processo de credenciamento para se tornar HAC no sul do Brasil [18], corroborando a importância da acreditação em HAC para melhorar não apenas a amamentação oportuna, mas também a duração do aleitamento materno exclusivo entre recém-nascidos saudáveis [19] e aqueles que precisam de cuidados intensivos de tratamento [20], assim como para reduzir o uso de chupeta [21].

Embora a OMS recomende uma taxa de cesariana de 10% [22], o nosso estudo encontrou uma taxa de 45%. Esta taxa foi diferente entre os HAC (32,1%) e não HAC (50,7%), o que pode ser explicado por um critério adicional estabelecido pelo Ministério da Saúde para certificação como Hospital Amigo da Criança (além dos requisitos da UNICEF/OMS em conformidade com os 10 passos): a redução das taxas de cesariana [23]. No estudo "Nascer no Brasil", as crianças nascidas por parto vaginal tiveram quase três vezes mais chance de serem amamentadas na primeira hora após o nascimento do que os nascidos por cesariana. Isto é consistente com os resultados de um estudo realizado no Rio de Janeiro, onde a cesariana reduziu pela metade a prevalência da amamentação na primeira hora após o nascimento [8], e com os achados de uma revisão sistemática, onde a cesariana foi o fator mais freqüentemente associado de forma negativa ao início oportuno da amamentação [7], já que os procedimentos e rotinas pós cesariana podem atrasar o contato precoce entre mãe e bebê.

Moreira *et al.* [24] relataram um efeito sinérgico entre o parto vaginal e o nascimento em Hospital Amigo da Criança, já que ambos representam boas práticas hospitalares para o recém-nascido. Supomos que a cesariana não deve representar um risco para o início oportuno da amamentação se ambos, recém-nascidos e as mães, estão em boas condições e se a cirurgia ocorrer após o início do trabalho de parto, o que pode indicar maturidade do recém-nascido para iniciar a amamentação. No entanto, a taxa de cesariana no Brasil é significativamente maior no setor privado, com cerca de 80% de cesarianas realizadas sem a mãe ter iniciado trabalho de parto [25].

Recém-nascidos prematuros e de baixo peso tiveram metade da chance de serem amamentados na primeira hora após o nascimento. Isto pode ser devido a rotinas e intervenções desnecessárias (oxigenioterapia, aspiração de vias aéreas superiores, entre outros) que podem desnecessariamente separar mãe e filho na sala de parto [24]. Nosso estudo incluiu apenas recém-nascidos prematuros com mais de 32 0/7 semanas de gestação e crianças com peso baixo ao nascer a partir de 1500 gramas, condições que, embora possam inspirar cuidados, podem não ser uma barreira para a amamentação na primeira hora após o nascimento. É importante melhorar tanto a prevenção da prematuridade, como os cuidados neonatais para as crianças mais vulneráveis [26], já que outro estudo também mostrou a prematuridade como fator de risco para a amamentação na primeira hora após o nascimento [27] .

Mães que recebem informações sobre aleitamento materno durante o pré-natal tiveram maiores chances para amamentar seus bebês na primeira hora após o nascimento, semelhante ao resultado de um estudo realizado na Bahia [27] (Vieira, 2010), mostrando a importância do pré-natal para o início da amamentação.

Considerando fatores distais, apenas idade materna e região de residência foram associadas com o resultado. Um estudo no sul do Brasil também encontrou associação entre a idade da mãe acima de 34 anos e menores chances de amamentação na primeira hora após o nascimento [18]. Este resultado pode ser devido a um efeito de coorte, já que mães mais velhas eram menos expostas à prática crescente de aleitamento materno no Brasil [28]. Quanto à região de residência, uma pesquisa nacional realizada em 2006 [28] e um estudo nas capitais brasileiras em 2008 [13] evidenciaram uma maior prevalência de aleitamento materno na primeira hora, na região Norte do Brasil, o que pode ser devido a fatores culturais, já que a maioria da população indígena está concentrada nesta região.

**Conclusões**:

Os fatores proximais estudados foram os mais fortemente associados à amamentação oportuna, trazendo evidências sobre a importância de adotar a Iniciativa Hospital Amigo da Criança para melhorar as práticas perinatais e o início oportuno da amamentação. Especial atenção deve ser dada à associação negativa entre cesariana sem indicação clínica e a amamentação na primeira hora após o nascimento, trazendo mais evidências para os esforços do governo para diminuir esta prática nociva no Brasil. A prematuridade e o baixo peso ao nascer são fatores difíceis de serem modificados, mas ganhos no acesso e qualidade dos cuidados pré-natais poderiam contribuir para um declínio na sua prevalência e para melhorar as taxas de amamentação oportuna. Recomendamos esforços na implementação da IHAC, estendendo-se esta iniciativa ao setor privado. Esta medida poderia contribuir não só para melhorar as taxas de amamentação oportuna, mas também para reduzir as cesarianas desnecessárias.

**Referências**

1. Edmond KM, Zandoh C, Quigley MA, Amenga-Etego S, Owusu-Agyei S, Kirkwood BR. Delayed breastfeeding initiation increases risk of neonatal mortality. Pediatrics. 2006; 117(3): e380-6. DOI:10.1542/peds.2005-1496.
2. Boccolini CS, Carvalho ML, Oliveira MIC, Pérez-Escamilla R. Breastfeeding during the first hour of life and neonatal mortality. J Pediatr (Rio J). 2013; 89(2):131-136.
3. Anderson GC, Moore E, Hepworth J, Bergman N. Early skin to skin contact for mothers and their healthy newborn infants. Cochrane Database Sist Rev. 2007; (3):CD003519.
4. Bystrova K, Ivanova V, Edhborg M, Matthiesen AS, Ranjsö-Arvidson AB, Mukhamedrakhimov R, et al. Early contact versus separation: effects on mother–infant interaction one year later. Birth. 2009; 36(2):97-109.
5. Albesharat R, Ehrmann MA, Korakli M, Yazaji S, Vogel RF. Phenotypic and genotypic analyses of lactic acid bacteria in local fermented food, breast milk and faeces of mothers and their babies. Syst Appl Microbiol. 2011;34:148-55.
6. [Ballard O](http://www.ncbi.nlm.nih.gov/pubmed/?term=ballard%25252525252520o%2525252525255bauthor%2525252525255d&cauthor=true&cauthor_uid=23178060), [Morrow AL](http://www.ncbi.nlm.nih.gov/pubmed/?term=morrow%25252525252520al%2525252525255bauthor%2525252525255d&cauthor=true&cauthor_uid=23178060). Human Milk Composition: Nutrients and Bioactive Factors. [Pediatr Clin North Am.](http://www.ncbi.nlm.nih.gov/pubmed/23178060) 2013; 60(1):49-74
7. Esteves TMB, Daumas RP, Oliveira MIC, Andrade CAF, Leite IC. Factors associated to breastfeeding in the first hour of life: systematic review. Rev. Saude Publica [online]. 2014; 48(4): 697-708.
8. Boccolini CS, Carvalho ML, Oliveira MIC, Vasconcellos AGG. Factors associated with breastfeeding in the first hour of life. Rev Saúde Pública. 2011; 45(1).
9. Leal MC, Pereira APE, Domingues RMSM, Theme Filha MM, Dias MAB, Nakamura-Pereira M et al. Obstetric interventions during labor and childbirth in Brazilian low-risk women. Cad. Saúde Pública 2014; 30, suppl.1: S17-S32.
10. Leal MC, Silva AAM, Dias MAB, Gama SGN, Rattner D, Moreira ME, et al. Birth in Brazil: national survey into labour and birth. Reproductive Health 2012; 9:15.
11. Dias MAB, Domingues RMSM, Schilithz AOC, Nakamura-Pereira M, Diniz CSG, Brum IR, et al. Incidence of maternal near miss in hospital childbirth and postpartum: data from the Birth in Brazil study. Cad. Saude Publica. 2014; 30 Sup:S169-S181.
12. [Victora CG](http://www.ncbi.nlm.nih.gov/pubmed/?term=victora%25252525252520cg%2525252525255bauthor%2525252525255d&cauthor=true&cauthor_uid=9126524), [Huttly SR](http://www.ncbi.nlm.nih.gov/pubmed/?term=huttly%25252525252520sr%2525252525255bauthor%2525252525255d&cauthor=true&cauthor_uid=9126524), [Fuchs SC](http://www.ncbi.nlm.nih.gov/pubmed/?term=fuchs%25252525252520sc%2525252525255bauthor%2525252525255d&cauthor=true&cauthor_uid=9126524), [Olinto MT](http://www.ncbi.nlm.nih.gov/pubmed/?term=olinto%25252525252520mt%2525252525255bauthor%2525252525255d&cauthor=true&cauthor_uid=9126524). The role of conceptual frameworks in epidemiological analysis: a hierarchical approach. [Int J Epidemiol.](http://www.ncbi.nlm.nih.gov/pubmed/9126524) 1997; 26(1):224-7.
13. Ministério da Saúde. II Pesquisa de Prevalência de Aleitamento Materno nas Capitais Brasileiras e Distrito Federal. Brasília, DF: Editora do Ministério da Saúde, 2009.
14. Rea MF. Reflexões sobre a amamentação no Brasil: de como passamos a 10 meses de duração. Cad Saude Publica 2003;19 Suppl 1:S37-45.
15. World Health Organization. Baby-friendly hospital initiative: revised, updated and expanded for integrated care. Section 2. Strengthening and sustaining the baby-friendly hospital initiative: a course for decision-makers. Geneva; 2009.
16. Righard L, Alade M. Effect of delivery room routines on success of first breast-feed. Lancet. 1990; 336: 1105-07.
17. Araújo MF, Schmitz BA. Twelve years of the Baby-Friendly Hospital Initiative in Brazil. [Rev Panam Salud Publica](http://www.ncbi.nlm.nih.gov/pubmed/17976275) 2007; 2(2): 91-9.
18. Silveira RB, Albernaz E, Zuccheto LM. Factors associated with the initiation of breastfeeding in a city in the south of Brazil. Rev. Bras. Saude Mater. Infant. 2008; 8(1): 35-43.
19. Abrahams SW, Labbok MH. Exploring the impact of the Baby-Friendly Hospital Initiative on trends in exclusive breastfeeding. International Breastfeeding Journal [online]. 2009; 4:11.
20. Vannuchi MTO, Monteiro CA, Rea MF, Andrade SM, Matsuo T. The Baby-Friendly Hospital Initiative and breastfeeding in a neonatal unit. Rev. Saude Publica [online]. 2004; 38(3): 422-428.
21. Venancio SI, Saldiva SRDM, Escuder MML, Giugliani ERJ. The Baby-Friendly Hospital Initiative shows positive effects on breastfeeding indicators in Brazil. J Epidemiol Community Health 2012; 66:914-918.
22. Ye J, Betrán AP, Guerrero Vela M, Souza JP, Zhang J. Searching for the optimal rate of medically necessary cesarean delivery. Birth Berkeley Calif. 2014; 41(3):237–244.
23. Ortiz PN, Rolim RB, Souza MFL, Soares PL, Vieira TO, Vieira GO, et al. Comparing breast feeding practices in baby friendly and non-accredited hospitals in Salvador, Bahia. Rev Bras Saude Mater Infant. 2011; 11(4): 405-413.
24. Moreira MEL, Gama SGN, Pereira APE, Silva AAM, Lansky S, Pinheiro RS, et al. Clinical practices in the hospital care of healthy newborn infant in Brazil. Cad Saude Publica 2014; 30 Sup: S128-S139.
25. Domingues RMSM, Dias MABD, Nakamura-Pereira M, Alves JT , d’Orsi E, Pereira APE, et al. Process of decision-making regarding the mode of birth in Brazil: from the initial preference of women to the final mode of birth Cad. Saude Publica 2014; 30 Sup:S101-S116.
26. Lansky S, Friche AAL, Silva AAM, Campos D, Bittencourt DAS, Carvalho ML, et al. Birth in Brazil survey: neonatal mortality, pregnancy and childbirth quality of care. Cad. Saude Publica 2014; 30 Sup: S192-S207.
27. Vieira TO, Vieira GO, Giugliani ERJ, Mendes CMC, Martins CC, Silva LR. Determinants of breastfeeding initiation within the first hour of life in a Brazilian population: cross-sectional study. BMC Public Health 2010; 10:760.
28. Ministério da Saúde. Pesquisa Nacional de Demografia e Saúde da Criança e da Mulher. Brasília, DF. Ed. Ministério da Saúde; 2008.

**Lista de Abreviações:**

**IHAC:** Iniciativa Hospital Amigo da Criança

**HAC:** Hospital Amigo da Criança

**ENSP:** Escola Nacional de Saúde Pública

**FIOCRUZ:** Fundação Oswaldo Cruz

**HIV:** Vírus da Imunodeficiência Humana

**APGAR:** alguns parâmetros avaliados nos primeiros cinco minutos de vida: A – Tônus muscular; P – Frequência Cardíaca; G – Irritabilidade reflexa; A – Cor da pele; R – Respiração. .

**OR –** Odds Ratio

**IC –** Intervalo de Confiança

**OMS –** Organização Mundial de Saúde

**UNICEF-** Fundo das Nações Unidas para a infância.

**Conflitos de interesse:**

Os autores declaram que não têm conflitos de interesse.

**Contribuições dos autores:**

MLC, CSB, MICO e MCL participaram da concepção e desenho do estudo, análise e interpretação dos dados, elaboração e revisão do manuscrito, e são responsáveis por todas as informações fornecidas. Todos os autores leram e aprovaram o manuscrito final.

**Agradecimentos:**

Apoio Financeiro

Esta pesquisa recebeu apoio financeiro do Conselho Nacional de Desenvolvimento Científico e Tecnológico (CNPq) e da Escola Nacional de Saúde Pública – Fiocruz, Brasil.

**Lista de tabelas:**

Tabela 1: Prevalência de características maternas, do recém-nascido e do hospital, Brasil, 2011.

Tabela 2: Aleitamento materno na primeira hora de vida, conforme caraterísticas maternas, dos recém-nascidos e do hospital, Brasil, 2011.

Tabela 3: Fatores associados com aleitamento materno na primeira hora de vida, Brasil,

2011.

**Lista de Figuras**:

Figura 1: Modelo Teórico Hierarquizado para análise da amamentação na primeira hora de vida.

Tabela 1:Prevalência de características maternas, do recém-nascido e do hospital, Brasil, 2011

| **Variáveis/categorias** | n^a^ | %^b^ | IC 99% ^b^ |
| --- | --- | --- | --- |
| **Idade materna** |  |  |  |
| 12 - 19 anos | 4170 | 18,8 | 17,7-20,1 |
| 20 - 34 anos | 15454 | 70,9 | 69,7-72,1 |
| 35 anos ou mais | 2232 | 10,2 | 9,4-11,1 |
| Perdas | 5 |  |  |
| **Cor da pele/raça^c^** |  |  |  |
| Branca | 7486 | 34,3 | 31,9-36,9 |
| Preta | 1835 | 8,4 | 7,3-9,7 |
| Parda | 12150 | 55,7 | 53,3-58,2 |
| Amarela | 233 | 1,1 | 0,8-1,5 |
| Indígena | 91 | 0,4 | 0,2-0,7 |
| Perdas | 4 |  |  |
| **Escolaridade materna (anos) ^d^** |  |  |  |
| <= 7 | 5572 | 25,7 | 23,8-27,7 |
| 8 a 10 | 5566 | 25,7 | 24,4-27,0 |
| 11 a 14 | 88556 | 39,4 | 37,1-41,8 |
| 15 ou mais | 2001 | 9,2 | 7,8-10,9 |
| Perdas | 103 |  |  |
| **Trabalho materno** |  |  |  |
| Não | 12885 | 59,1 | 57,4-60,8 |
| Sim | 8913 | 40,9 | 39,2-42,6 |
| Perdas | 1 |  |  |
| **Estado civil (no momento do parto)** |  |  |  |
| Sem companheiro | 3984 | 18,3 | 17,2-19,4 |
| Com companheiro/casada | 17808 | 81,7 | 80,6-82,8 |
| Perdas | 6 |  |  |
| **Paridade** |  |  |  |
| Primípara | 11618 | 53,3 | 52,0-54,6 |
| Multípara | 10180 | 46,7 | 45,4-48,0 |
| Perdas | 1 |  |  |
| **Região de residência** |  |  |  |
| Norte | 2106 | 9,7 | 8,6-10,9 |
| Nordeste | 6160 | 28,3 | 26,0-30,6 |
| Sudeste | 9318 | 42,7 | 39,7-45,8 |
| Sul | 2775 | 12,7 | 11,5-14,0 |
| Centro-Oeste | 1440 | 6,6 | 5,5-7,9 |
| Perdas | 0 |  |  |
| **Desejo de engravidar** |  |  |  |
| Sim | 9619 | 44,4 | 43,0-45,8 |
| Não por enquanto | 5619 | 25,9 | 24,7-27,2 |
| Não | 6420 | 29,6 | 28,2-31,1 |
| Perdas | 140 |  |  |
| **Financiamento do pré-natal** |  |  |  |
| Não fez pré-natal | 233 | 1,1 | 0,8-1,4 |
| Público | 15065 | 69,3 | 67,1-71,4 |
| Misto | 825 | 3,8 | 3,3-4,4 |
| Privado | 5626 | 25,9 | 24,0-27-9 |
| Perdas | 49 |  |  |
| **Informações sobre aleitamento materno no pré-natal** |  |  |  |
| Não fez pré-natal | 233 | 1,1 | 0,8-1,4 |
| Sim | 13848 | 35,0 | 33,1-36,9 |
| Não | 7624 | 63,5 | 61,6-65,4 |
| Perdas | 93 |  |  |
| **Fumou durante a gravidez** |  |  |  |
| Não | 19712 | 90,5 | 89,6-91,3 |
| Sim, parte da gestação | 607 | 2,8 | 2,4-3,3 |
| Sim, toda gestação | 1467 | 6,7 | 6,1-7,4 |
| Perdas | 13 |  |  |
| **Financiamento do parto** |  |  |  |
| Público | 17272 | 79,2 | 77,2-81,1 |
| Privado | 4527 | 20,8 | 18,9-22,8 |
| Perdas | 0 |  |  |
| **Hospital Amigo da Criança ^e^** |  |  |  |
| Sim | 7159 | 32,8 | 25,7-40,9 |
| Em credenciamento | 1571 | 7,2 | 3,7-13,5 |
| Não | 13068 | 60,0 | 51,9-67,5 |
| Perdas | 0 |  |  |
| **Acompanhante no hospital** |  |  |  |
| Nenhum | 5201 | 23,9 | 20,0-28,2 |
| Tempo parcial | 12171 | 55,9 | 51,3-60,3 |
| Todo o tempo | 4418 | 20,3 | 16,5-24,6 |
| Perdas | 8 |  |  |
| **Tipo de parto** |  |  |  |
| Normal | 10249 | 47,0 | 44,0-50,0 |
| Fórceps | 312 | 8,1 | 6,9-9,4 |
| Cesariana intraparto | 1766 | 43,5 | 40,9-46,1 |
| Cesariana anteparto | 9472 | 1,4 | 0,9-2,3 |
| Perdas | 0 |  |  |
| **Idade gestacional (semanas)** |  |  |  |
| 32 0/7 a 36 6/7 | 1905 | 8,7 | 7,7-10,0 |
| 37 0/7 | 19894 | 91,3 | 90,0-92,3 |
| Perdas | 0 |  |  |
| **Peso ao nascer** |  |  |  |
| 2500g ou mais | 20181 | 93,4 | 92,5-94,2 |
| 1500-2499g | 1422 | 6,6 | 5,8-7,5 |
| Perdas | 195 |  |  |

a – Amostra final de mães que responderam ao questionário da pesquisa “ Nascer Brasil” em 2011 e que atenderam aos critérios do estudo – casos sem correção por peso amostral;

b - Prevalência e Intervalo de Confiança de 99% (IC 99%) da amostra final válida, considerando o desenho complexo da amostra;

c - Cor da pele ou raça auto referida, conforme classificação do Instituto Brasileiro de Geografia e Estatística, 2010;

d - Escolaridade conforme classificação do Instituto Brasileiro de Geografia e Estatística, 2010;

e – Baseado em informações coletadas com o gerente do hospital.

Tabela 2: Aleitamento materno na primeira hora de vida, conforme caraterísticas maternas, dos recém-nascidos e do hospital, Brasil, 2011.

| **Variáveis/categorias** | Prevalência^a^ | Prevalência IC 99% ^a^ | OR Bruta ^b^ | OR Bruta IC 99% ^b^ | |
| --- | --- | --- | --- | --- | --- |
| **Idade materna** |  |  |  |  | |
| 12 - 19 anos | 62,5 | 58,2-66,6 | 2,01 | 1,60-2,52 | |
| 20 - 34 anos | 55,8 | 51,8-59,8 | 1,52 | 1,29-1,81 | |
| 35 anos ou mais | 45,3 | 40,1-50,7 | 1,00 | - | |
| **Cor da pele/raça^c^** |  |  |  | |  |
| Branca | 51,4 | 46,6-56,2 | 1,00 | | - |
| Preta | 59,4 | 53,6-64,9 | 1,38 | | 1,07-1,79 |
| Parda | 58,1 | 54,0-62,1 | 1,31 | | 1,11-1,55 |
| Amarela | 62,6 | 48,7-74,7 | 1,58 | | 0,90-2,78 |
| Indígena | 75,7 | 53,4-89,8 | 2,94 | | 1,03-8,41 |
| **Escolaridade materna (anos) ^d^** |  |  |  | |  |
| <= 7 | 62,8 | 58,7-66,7 | 2,72 | | 2,08-3,55 |
| 8 a 10 | 61,4 | 57,1-65,7 | 2,57 | | 1,96-3,36 |
| 11 a 14 | 52,2 | 47,6-56,9 | 1,76 | | 1,41-2,19 |
| 15 ou mais | 38,3 | 32,6-44,3 | 1,00 | | - |
| **Trabalho materno** |  |  |  | |  |
| Não | 60,0 | 56,2-63,8 | 1,49 | | 1,31-1,70 |
| Sim | 50,2 | 45,8-54,6 | 1,00 | | - |
| **Estado civil (no momento do parto)** |  |  |  | |  |
| Sem companheiro | 59,0 | 54,3-63,4 | 1,16 | | 1,02-1,31 |
| Com companheiro/casada | 55,4 | 51,5-59,1 | 1,00 | | - |
| **Paridade** |  |  |  | |  |
| Primípara | 58,6 | 54,7-62,4 | 1,00 | | - |
| Multípara | 53,1 | 48,9-57,3 | 1,25 | | 1,11-1,50 |
| **Região de residência** |  |  |  | |  |
| Norte | 71,0 | 65,3-76,1 | 2,30 | | 1,56-3,39 |
| Nordeste | 53,7 | 47,6-59,7 | 1,09 | | 0,75-1,59 |
| Sudeste | 51,5 | 44,4-58,6 | 1,00 | | - |
| Sul | 61,1 | 50,7-70,6 | 1,48 | | 0,89-2,47 |
| Centro-Oeste | 63,1 | 55,7-70,0 | 1,61 | | 1,06-2,46 |
| **Desejo de engravidar** |  |  |  | |  |
| Sim | 54,0 | 49,9-58,1 | 1,00 | | - |
| Não por enquanto | 56,7 | 52,2-61,1 | 1,17 | | 0,98-1,26 |
| Não | 58,6 | 54,2-62,7 | 1,20 | | 1,04-1,40 |
| **Fumou durante a gravidez** |  |  |  | |  |
| Não | 55,6 | 51,8-59,3 | 1,00 | | - |
| Sim, parte da gestação | 57,3 | 49,0-65,3 | 1,07 | | 0,81-1,43 |
| Sim, toda gestação | 61,0 | 54,7-66,9 | 1,25 | | 1,01-1,55 |
| **Financiamento do pré-natal** |  |  |  | |  |
| Não fez pré-natal | 52,8 | 40,2-65,0 | 1,71 | | 0,98-2,99 |
| Público | 62,4 | 58,3-66,3 | 2,55 | | 1,97-3,29 |
| Misto | 52,7 | 45,3-60,0 | 1,71 | | 1,23-2,38 |
| Privado | 39,5 | 33,7-45,6 | 1,00 | | - |
| **Informações sobre aleitamento materno no pré-natal** |  |  |  | |  |
| Não fez pré-natal | 52,8 | 40,2-65,0 | 1,02 | | 0,61-1,71- |
| Sim | 58,2 | 54,5-61,8 | 1,28 | | 1,11-1,48 |
| Não | 52,2 | 47,2-57,0 | 1,00 | | - |
| **Financiamento do parto** |  |  |  | |  |
| Público | 61,1 | 57,0-65,1 | 2,73 | | 1,94-3,86 |
| Privado | 36,5 | 29,4-44,3 | 1,00 | | - |
| **Hospital Amigo da Criança ^e^** |  |  |  | |  |
| Sim | 69,4 | 64,9-73,6 | 2,49 | | 1,85-3,34 |
| Em credenciamento | 63,9 | 51,0-75,1 | 1,94 | | 1,11-3,41 |
| Não | 47,7 | 42,5-53,0 | 1,00 | | - |
| **Acompanhante no hospital** |  |  |  | |  |
| Nenhum | 55,6 | 49,8-61,2 | 1,00 | | - |
| Tempo parcial | 54,3 | 49,8-58,8 | 0,95 | | 0,75-1,20 |
| A todo tempo | 61,1 | 55,4-66,6 | 1,26 | | 0,92-1,72 |
| **Tipo de parto** |  |  |  | |  |
| Normal | 70,3 | 66,2-74,1 | 3,29 | | 2,67-4,06 |
| Fórceps | 60,3 | 47,9-71,5 | 2,11 | | 1,27-3,51 |
| Cesariana intraparto | 48,7 | 43,0-54,4 | 1,32 | | 1,07-1,63 |
| Cesariana anteparto | 41,8 | 37,2-46,5 | 1,00 | | - |
| **Idade gestacional (semanas)** |  |  |  | |  |
| 32 0/7 a 36 6/7 | 37,4 | 32,9-42,1 | 0,44 | | 0,36-0,53 |
| 37 0/7 | 57,8 | 53,9-61,6 | 1,00 | | - |
| **Peso ao nascer** |  |  |  | |  |
| 2500g ou mais | 57,4 | 53,5-61,2 | 0,42 | | 0,36-0,50 |
| 1500-2499g | 36,3 | 31,0-42,0 | 1,00 | | - |
| **Total** | **56,0** | **52,2-59,7** |  | |  |

a –Prevalência e Intervalo de Confiança de 99% (IC 99%) da mostra final válida, considerando o desenho complexo da amostra;

b – Odds Ratio (OR) não ajustada e Intervalo de Confiança de 99% (IC 99%), considerando o desenho complexo da amostra;

c - Cor da pele ou raça auto referida, conforme classificação do Instituto Brasileiro de Geografia e Estatística, 2010;

d - Escolaridade conforme classificação do Instituto Brasileiro de Geografia e Estatística, 2010;

e – Baseado em informações coletadas com o gerente do hospital.

Tabela 3: Fatores associados com aleitamento materno na primeira hora de vida, Brasil, 2011.

| **Variáveis/categorias** | OR ^a^ | Invervalo Confiança 99% ^a^ |
| --- | --- | --- |
| **Idade materna** |  |  |
| 12 - 19 anos | 1,33 | (1,02-1,74) |
| 20 - 34 anos | 1,23 | (1,01-1,50) |
| 35 anos ou mais | 1,00 | - |
| **Escolaridade maternal (anos) ^b^** |  |  |
| <= 7 | 1,01 | (0,76-1,36) |
| 8 a 10 | 1,05 | (0,82-1,35) |
| 11 a 14 | 0,99 | (0,78-1,26) |
| 15 ou mais | 1,00 | - |
| **Paridade** |  |  |
| Primípara | 0,85 | (0,70-1,03) |
| Multípara | 1,00 | - |
| **Região de residência** |  |  |
| Norte | 1,81 | (1,19-2,76) |
| Nordeste | 0,92 | (0,63-1,35) |
| Sudeste | 1,43 | (0,83-2,46) |
| Sul | 1,37 | (0,81-2,32) |
| Centro-Oeste | 1,00 | - |
| **Financiamento do pré-natal** |  |  |
| Não fez pré-natal | 0,96 | (0,20-4,74) |
| Público | 1,33 | (1,01-1,76) |
| Misto | 1,35 | (0,92-1,99) |
| Privado | 1,00 | - |
| **Informações sobre aleitamento materno no pré-natal** |  |  |
| Não fez pré-natal | 1,03 | (0,30-3,56) |
| Sim | 1,38 | (1,17-1,63) |
| Não | 1,00 | - |
| **Hospital Amigo da Criança ^c^** |  |  |
| Sim | 2,07 | (1,50-2,86) |
| Em credenciamento | 1,44 | (0,71-2,93) |
| Não | 1,00 | - |
| **Tipo de parto** |  |  |
| Normal | 2,81 | (2,19-3,62) |
| Fórceps | 2,11 | (1,30-3,44) |
| Cesariana intraparto | 1,18 | (0,94-1,48) |
| Cesariana anteparto | 1,00 | - |
| **Idade gestacional (semanas)** |  |  |
| 32 0/7 a 36 6/7 | 0,47 | (0,38-0,59) |
| 37 0/7 | 1,00 | - |
| **Peso ao nascer** |  |  |
| 2500g ou mais | 0,51 | (0,39-0,66) |
| 1500-2499g | 1,00 | - |

a – Odds Ratio (OR) ajustada e Intervalo de Confiança de 99% (IC 99%), obtidos de modelo de regressão logística, e considerando o desenho complexo da amostra;

b - Escolaridade conforme classificação do Instituto Brasileiro de Geografia e Estatística, 2010;

c – Baseado em informações coletadas com o gerente do hospital.

Figura 1: Modelo Teórico Hierarquizado para análise da amamentação na primeira hora de vida

**Amamentação na primeira hora de vida**

**Nível intermediário**

**Características da Gestação**

- Desejo de engravidar

- Fumo durante a gestação

**Cuidado Pré-natal**

- Financiamento pré-natal

- Informação sobre aleitamento na primeira hora de vida

**Nível distal**

**Características Maternas**

- Idade

- Cor da pele/ raça

- Anos de escolaridade

- Trabalho

- Estado civil

- Paridade

**Características Familiares**

- Região de residência

**Nível proximal**

**Características do Parto**

**-** Financiamento Hospitalar

- Hospital Amigo da Criança

- Acompanhante no hospital

**-** Tipo de parto

**Características do Recém-nato**

- Idade Gestacional

- Peso ao Nascer
