# Supplementary material for: The baby-friendly hospital initiative and breastfeeding at birth in Brazil: a cross sectional study
Source: Reprod Health. 2016 Oct 17;13(Suppl 3):119. doi: 10.1186/s12978-016-0234-9 (PMC5073809; doi:10.1186/s12978-016-0234-9)
Supplement: Additional file 2: — Peer review. (DOCX 28 kb) [file 12978_2016_234_MOESM2_ESM.docx]

**Referee’s comments to the authors– this sheet WILL be seen by the author(s) and published with the article**

| Title | The baby-friendly hospital initiative and breastfeeding at birth in Brazil: a cross sectional study |
| --- | --- |
| Author(s) | Márcia Lazaro Carvalho; Cristiano Siqueira Boccolini; Maria Inês Couto de Oliveira and Maria do Carmo Leal |
| Referee’s name | Wendy Oddy |

**When assessing the work, please consider the following points, where applicable:**

1. Is the question posed by the authors new and well defined?
2. Are the methods appropriate and well described, and are sufficient details provided to replicate the work?
3. Are the data sound and well controlled?
4. Does the manuscript adhere to the relevant standards for reporting and data deposition?
5. Are the discussion and conclusions well balanced and adequately supported by the data?
6. Do the title and abstract accurately convey what has been found?
7. Is the writing acceptable?

Please make your report as constructive and detailed as possible in your comments so that authors have the opportunity to overcome any serious deficiencies that you find and please also divide your comments into the following categories:

- Major Compulsory Revisions (which the author must respond to before a decision on publication can be reached)
- Minor Essential Revisions (such as missing labels on figures, or the wrong use of a term, which the author can be trusted to correct)
- Discretionary Revisions (which are recommendations for improvement but which the author can choose to ignore)

Where possible please supply references to substantiate your comments.

When referring to the manuscript please provide specific page and paragraph citations where appropriate.

The Baby-Friendly Hospital Initiative and Breastfeeding at Birth in Brazil: a cross sectional study

The aim of this study was to analyze the association between delivery in a Baby-Friendly Hospital and timely initiation of breastfeeding. Data came from the Birth in Brazil survey, a nation-wide hospital based study of post-partum women and their newborns. It should be stated that the aim of the study was to compare breastfeeding duration with non-Baby Friendly Hospital births.

*We have modified the text:*

*‘Government policies are being developed in this direction, highlighting the accreditation of hospitals in the Baby-Friendly Hospital (BFH) initiative. The aim of this study was to analyze the association between delivery in a BFH (main exposure) compared to non BFH, and timely initiation of breastfeeding (outcome).’*

**Specific comments**

There is a need to be consistent with spelling of caesarean, as other places it is spelt caesarean throughout the manuscript. The issues with this manuscript are largely to do with errors in the English language.

*We have standardized the term ‘caesarean’ throughout the manuscript .*

**Abstract**

In results of the abstract, the ‘outcome’ is mentioned a number of times. However it is not clear what the outcome is. This needs to be specified clearly.

*We have added: ‘and timely initiation of breastfeeding (outcome).’ in the abstract.*

**Background**

Page 6 – line 3 – ‘the human milk’ should be ‘human milk’ only.

Page 6 – paragraph 3 – ‘Since the Baby-Friendly Hospital Initiative may play a key role to promote timely…’ should read ‘Since the Baby-Friendly Hospital Initiative may play a key role in promoting timely…’

*‘We have corrected according suggestions’*

**Methods**

Page 7- paragraph 1, line 2 –‘…births occurred in hospitals…which correspond to…’ should be ‘…births occurring in hospitals…which corresponds to…’

Page 7 – paragraph 1, line 4 – ‘…was coordinated by Oswaldo…’ should be ‘…was coordinated by **the** Oswaldo…’

*‘We have corrected according suggestions’*

Page 7 – paragraph 3 – of what relevance are ‘stillbirths’ in this manuscript? What are they included or mentioned here? Because a still birth is not a live birth, should they be excluded?

*We have excluded the term ‘stillbirth': The inclusion criteria to the “Birth in Brazil” survey was: hospital live births with gestational age of more than 22 weeks recorded in the medical file or weight greater than 500 grams. All miscarriages were excluded.*

Page 8 – 2^nd^ paragraph – is it correct that 924 mothers did not know about breastfeeding initiation of their infant?

*Unfortunately, yes, most of these 924 mothers did not know the exact time of birth and at what time they had breastfed, so they could not estimate the time between birth and breastfeeding.*

Page 9 – paragraph 2 – lines 5/6 – currently read ‘did not achieved p<0.001… were maintained’. Should read ‘did not achieve p<0.001… were retained’

Page 9 – paragraph 1 or results, line 4 – reads ‘…did not finish the elementary schooling, more than half were primipare,…’ should read ‘…did not finish elementary schooling, more than half were primiparous…’

Page 9 – paragraph 4 – ‘…we found association…’ should read ‘…we found associations…’

Page 10 – paragraph 1 line 2 and 4 – reads ‘…we have found association with…. The proximal variables associated to the outcome were…’ should read ‘…we found associations with…. The proximal variables associated with the outcome were…’

Page 10, paragraph 2 – line 1 reads ‘…mothers with higher chances to breastfed at the first hour of life…’ should read ‘…mothers with a higher chance of breastfeeding in the first hour of life…’

And page 10, paragraph 2, lines 5/6, reads ‘…also had higher chances to timely initiate breastfeeding….lower chances …’ should read ‘…also had higher odds of initiating breastfeeding in a timely manner….lower odds …’

*‘We have corrected according suggestions’*

**Discussion**

Page 12 – paragraph 2 – dis this study include ‘term’ infants, as authors state here that the study only included premature newborns with 32 0/7 to 36 6/7 gestational weeks…’

*‘We have included all children with gestational age > 32 weeks, as stated in methods’.*

*‘The inclusion criteria to the “Birth in Brazil” survey were: hospital live births; in which the stillbirth had a gestational age of more than 22 weeks recorded in the medical file or weight greater than 500 grams.’*

*And*

*‘ (…*) *we have established the following exclusion criteria: (…); and/or gestational age < 32 weeks’*

Throughout the manuscript the word ‘chances’ is used instead of ‘odds’.

Page 13 – paragraph 1 – 2^nd^ line – ‘…to the outcome’. Should read ‘…with the outcome’.

Page 13 – paragraph 1, last line reads ‘…since most of indigenous…’ should read ‘sine most of the indigenous…’

*‘We have corrected according suggestions’*

**Conclusions**

Page 13 – paragraph 2, line 2 – the word ‘evidences’ should be ‘evidence’; line 3 – ‘A special attention…’ should read ‘Special attention’; line 5 – ‘evidences’ should be ‘evidence’.

Authors contributions – fist line – ‘…participated on the study…’ should read ‘…participated in the study…’

*‘We have corrected according suggestions’*

**List of tables**

**Table 2: title ‘Breastfeeding at first hour of life…’ should read ‘‘Breastfeeding in the first hour of life…’**

**In Table 1 under ‘Intended pregnancy’, what does the ‘not for now’ category mean? What is the difference between ‘Not for now’ and ‘No’?**

*‘We changed for: “Did not intend to get pregnant now’ (she intended to get pregnant later, but not at that time), and “Not at all” means that she did not mean to get pregnant any more’.*

**On page 22 – under type of delivery, it looks like the numbers for Intrapartum caesarean and Antepartum caesarean are reversed – please check and change.**

*‘Thank you! We have corrected this information.’*

Table 2 should be labelled as ‘bivariate’ or ‘unadjusted’. In Table 2 under Birth-weight, commas are used as spacers whereas full stops are used everywhere else in this manuscript.

*‘We have added’:*

- ‘*Unadjusted factors associated with breastfeeding in the first hour after birth, according to mother, newborn and hospital characteristics, Brazil, 2011’.*

Table 3 title should reflect that it is reporting a multivariate analysis. Therefore include this in the table title. Why do the authors not just show the significant associations in table 3?

*‘We have excluded those variables from the table, adding the following note’:*

*“Adjusted Odds Ratio (AOR) and 99%CI, obtained from a logistic regression model and considering the complex sample design, adjusted for educational level and parity.”*

Also in table 3 under Gestational age, commas again have been used whereas full stops have been used everywhere else.

*‘We have corrected according suggestions’*

**Referee’s comments to the authors– this sheet WILL be seen by the author(s) and published with the article**

| Title | The baby-friendly hospital initiative and breastfeeding at birth in Brazil: a cross sectional study |
| --- | --- |
| Author(s) | Marcia Lázaro Carvalho; Cristiano Siqueira Boccolini; Maria Inês Couto de Oliveira and Maria do Carmo Leal |
| Referee’s name | Kajsa Brimdyr |

**When assessing the work, please consider the following points, where applicable:**

1. Is the question posed by the authors new and well defined?
2. Are the methods appropriate and well described, and are sufficient details provided to replicate the work?
3. Are the data sound and well controlled?
4. Does the manuscript adhere to the relevant standards for reporting and data deposition?
5. Are the discussion and conclusions well balanced and adequately supported by the data?
6. Do the title and abstract accurately convey what has been found?
7. Is the writing acceptable?

Please make your report as constructive and detailed as possible in your comments so that authors have the opportunity to overcome any serious deficiencies that you find and please also divide your comments into the following categories:

- Major Compulsory Revisions (which the author must respond to before a decision on publication can be reached)
- Minor Essential Revisions (such as missing labels on figures, or the wrong use of a term, which the author can be trusted to correct)
- Discretionary Revisions (which are recommendations for improvement but which the author can choose to ignore)

Where possible please supply references to substantiate your comments.

When referring to the manuscript please provide specific page and paragraph citations where appropriate.

**General comments:**

**This is an important and relevant study that will impact future decisions on a governmental and global scale. This research illustrates Brazil’s commitment to insuring public health through promotion, protection and support of breastfeeding. The size and scale of this study underscore the national commitment to breastfeeding, highlighting Brazil’s ability to both scale up and sustain breastfeeding programs. It is compelling to see Brazil’s commitment of resouces to such an important understanding of national health.**

**Minor essential revisions:**

**There are grammatical issues throughout the paper that I have attempted to note. The paper will be stronger once they addressed.**

*Thank you,*

*We have corrected all the grammar/spelling modifications sent by pdf file, reviewing the grammar.*

*Regarding those two issues:
1 - We have adopted the terminology “the first hour after birth”*

*2- In Brazil we adopt the terminology “skin color/race” as an official classification. We have added the following phrase in the paper:*

*“ It is important to state that in Brazil we classify race/ethnicity not based on ancestry taxonomy, but based on self-reported skin color/race, according to the official Brazilian Statistic Institute ´Instituto Brasileiro de Geografia e Estatística’definitions, 2010.”*
